# Semiquantitative CT imaging as a tool in improving detection of ground glass patches in patients with COVID-19 pneumonia and for better follow-up

**DOI:** 10.1186/s43055-022-00862-5

**Published:** 2022-08-16

**Authors:** Marian Fayek Farid Kolta, Mahmoud Alaa Abd-Elrehim Abd-Elaal Abouheif, Mohammed Raafat Abd El-Mageed

**Affiliations:** Faculty of Medicine, Cairo University, Giza, Egypt

**Keywords:** COVID-19, CT, Ground glass opacity, Semiquantitative assessment

## Abstract

**Background:**

Coronavirus disease 2019 (COVID-19) is a global pandemic caused by severe acute respiratory syndrome coronavirus 2 influencing millions of people worldwide. It has clinical symptoms going from mild symptoms in about 80% of patients to a case mortality rate of about 2% in hospitalized patients associated with radiologic findings at chest CT which is showing multifocal bilateral ground glass opacities (GGO) and consolidative patches with subpleural and peri-bronchovascular predominant distribution. The role of chest CT in COVID-19 is very crucial, so this study hypothesized that increasing the accuracy and rapidity of CT in the detection of COVID-19-related pneumonia will offer rapid management and intervention of affected cases and gain better outcomes. The aim of this study is to offer and assess the ability of a software computer program in helping the radiologists in rapid detection of COVID-19 pneumonic criteria.

**Results:**

This cross-sectional study involved 73 patients with clinical symptoms and real-time polymerase chain reaction test positive results diagnosed as COVID-19. They were referred to perform chest CT; their CT images were sent to a separate workstation to be automated and processed through the COVID-19 detector, and compared the finding of the radiologist and the COVID-19 detector. The median number of lesions was 2 among the studied participants ranging from 1 to 12 lesions. The most common affected site of the lesions was the lower lobes. There was a significant strong agreement (*P* value < 0.001, kappa = 0.923) between the radiologist and the semiquantitative CT assessment in the detection of GGO among patients with COVID-19 pneumonia. Also, there were 6 patients who underwent follow-up by semiquantitative CT and radiologist; the median number of lesions was 1 among the studied participants ranging from 1 to 8 lesions. There was a significant strong agreement (*P* value = 0.001, Kappa = 0.856) between the radiologist and the semiquantitative CT assessment in the detection of GGO during follow-up among patients with COVID-19 pneumonia.

**Conclusions:**

The tested computer program can accurately detect COVID-19 pneumonia as it has better visualization in detecting GGO for diagnosing and following up on COVID-19 pneumonia.

## Background

COVID-19 pneumonia is a global pandemic caused by severe acute respiratory syndrome coronavirus-2 (SARS-CoV-2) influencing millions of people worldwide. It has clinical symptoms going from mild symptoms in about 80% of patients to a case mortality rate of about 2% in hospitalized patients. The clinical spectrum of COVID-19 varies from asymptomatic or forms to clinical conditions represented by respiratory failure that requires mechanical ventilation and support in an ICU, to systemic manifestations in terms of sepsis, septic shock, and multiple organ dysfunction syndromes [1].

Non-contrast computed tomography (CT) of the chest is generally used for evaluation of the disease severity and to guide clinical management. Typically, COVID-19 pneumonia presents with bilateral predominantly peripheral ground glass patches with or without consolidation, with some patches that may attain rounded morphology. The lower lobes and posterior portions are more frequently involved [2].

Huang et al. reported that severe patients on admission often presented with bilateral multiple lobular and subsegmental consolidation on their chest CT, while mild patients often presented bilateral GGO and subsegmental consolidation on their chest CT [3].

In the early stages, most of the patients showed more GGO with fewer lobes affection compared to the subsequent follow-up scans. Nevertheless, more lobes number affection, appearance of crazy-paving pattern and consolidative patches appeared in the majority of patients over time [4].

Semiquantitative method relies on measuring the Hounsfield unit (HU) of the lesion as well as it relays on the color-coded image assessment, while the quantitative method is an objective method depending only upon the calculation of HU values. Quantitative imaging analysis (QIA), which allows for accurate identification of lung tissue density by HU, can help in differentiating otherwise subtle radiographic diagnoses [5].

The combined use of visual and color-coded images improves the early detection of faint ground glass opacities seen in early COVID-19 stages with consequent improvement in the disease prognosis and limiting the spread of this highly contagious disease [5].

To improve the prognosis of the patients with severe NCP (who have a high risk of disease progression and higher incidence of mortality), the semiquantitative method with relatively higher sensitivity is more qualified to identify patients with severe type [6].

Semiquantitative method depends on measuring the HU unit of the lesion as well as it relays on the color-coded image assessment while the quantitative method is an objective method that depends only upon HU values calculation [5].

Recently, Mei et al. proposed an artificial intelligence system to help rapidly diagnose COVID-19 patients by integrating chest CT findings, clinical symptoms, exposure history and laboratory testing, which gives an innovative and timesaving application of deep, learning to public emergent events. Likewise, a deep learning framework, specifically for automatic segmentation of inflammatory lesion, should have a promising potential to maximally reduce labor time [6].

Up to date, semiquantitative CT method provides an important way to diagnose and evaluate COVID-19 disease. The obtained information derived by this method will provide quantitative information on inflammation allowing for better assessment of the clinical severity of the disease, monitoring disease progression, determining the efficacy of specific treatments and prediction of the disease prognosis. Semiquantitative CT imaging can help in the control and prevention of acute respiratory distress syndrome caused by COVID-19 infection [7].

The aim of this study is to offer and assess the ability of a software computer program in helping the radiologists in rapid detection of COVID-19 pneumonic criteria.

## Methods

This cross-sectional study involved 73 patients (53 females and 20 males), with the age range of 12–69 years. All patients with clinical symptoms such as fever, cough, sore throat, rhinorrhea, shortness of breath, etc., and positive PCR for COVID-19 were referred from the outpatient clinic of chest department in our institution to perform CT of lungs in the radiology department. The study was conducted in the period from February 2021 to June 2021.

Inclusion criteria

- All age-groups.
- Males or females.
- Symptoms of viral infection.
- Imaging manifestations of pneumonia.
- Positive PCR results.

Exclusion criteria

- Patients without chest CT thin sections study.
- Patients with positive PCR results but without pneumonia on chest CT.
- Chest CT with severe movement artifacts.

All patients were subjected to the following:

- Informed written consent prior to enrollment.
- Full medical history and examination.
- CT study examination with their CT images sent to a separate workstation to be automated and processed through the COVID-19 detector.

### Protocol for CT chest study

All patients performed CT scans at 16-multidetector CT (MDCT) scanner, SOMATOM, Emotion Siemens.

Patients were scanned during full inspiration in the supine position. No IV contrast injection was introduced.

Contiguous axial slices of CT scans were obtained at 5 mm intervals, with a 5 mm collimation, 120–130 Kvp, and at 125 mA. All images were obtained at window levels appropriate for lung parenchyma with multi-planar reformatted images performed.

### COVID-19 detector software (machine learning):

#### Establishing and training

Machine learning represents computer algorithms that can learn and improve automatically through iterated experience. Deep learning is a subset of machine learning which is responsible for the learning and training of artificial deep neural networks.

CT chest images are passed to the network, and after processing it decides whether the images are normal tissue or infected according to the numerical coding of the output value.

#### Prediction mechanism

The neural network implemented into the COVID-19 detector software has been designed and trained. The network was pre-trained on ImageNet and then on COVIDx-CT datasets. The COVID Net-CT network has been tailored to make one of the following three predictions: (a) no infection (normal), and (b) COVID-19 viral infection.

**Fig. 1:**
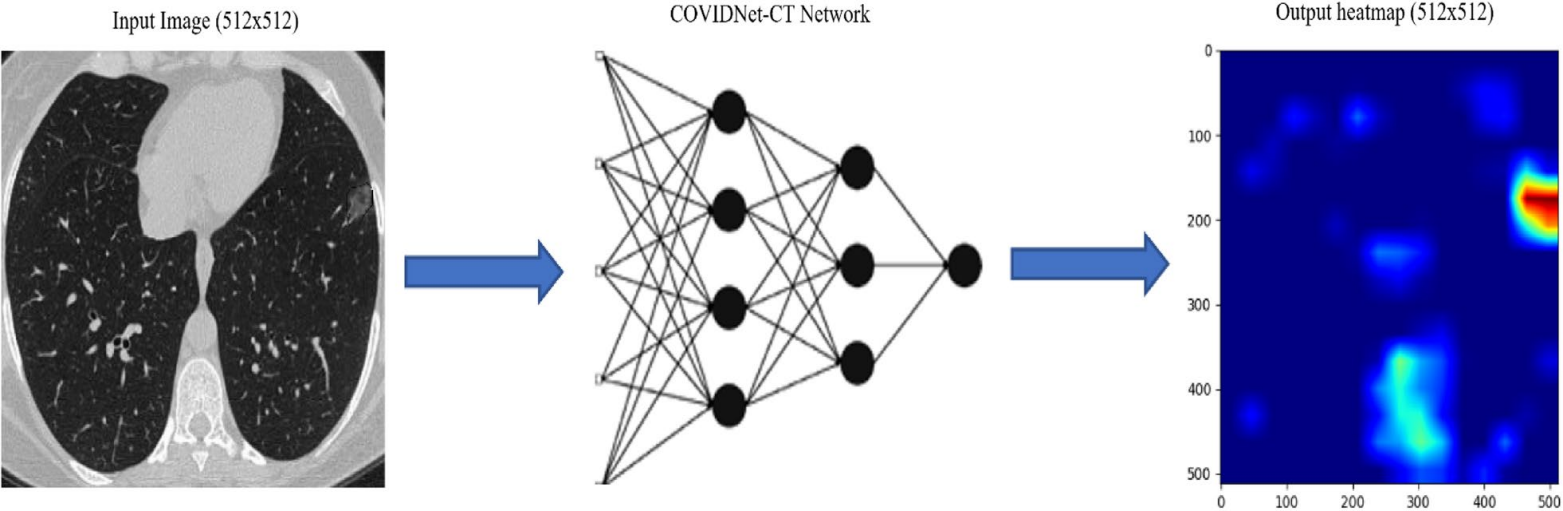
Data and prediction pipeline of the COVID-19 detector software

Furthermore, it performs the two-class prediction to each single pixel of the image, saying that to which class this pixel belongs and how much affiliation the pixel has to this class. All this information is compactly represented in the resulting heatmap as shown in Fig. 1.

#### Software architecture

**Fig. 2:**
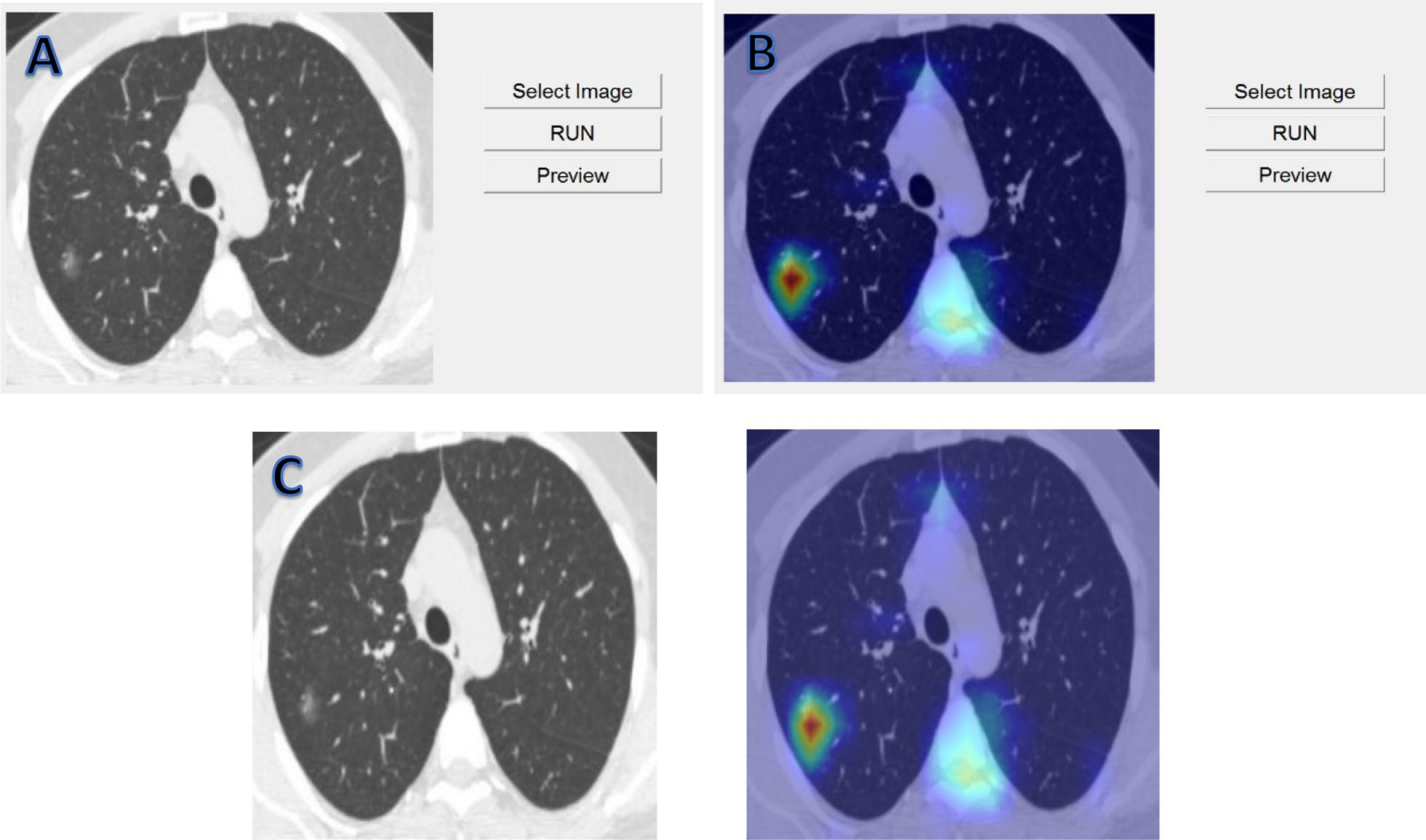
User interface of the COVID-19 detector software. **A** CT lung image is loaded into the software but not yet possessed. **B** When the program runs, the resulting heat map is displayed over the original image. **C** Original and output images displayed altogether after button preview is clicked

In order to be able to perform prediction on the CT lung images and effortlessly obtain understandable results in terms of prediction class and localization of the detected Covid19 patches, a Windows Desktop application (Fig. 2) has been built for this purpose.

The radiologist first selects the intended image anywhere from the operating PC or a local network and then simply clicks on RUN to let the program analyze the image and generate the result automatically. It takes the program few seconds to display the result because of the heavy computational load executed by the model. The predicted class, original and output images can be displayed altogether if the button Preview is clicked.

Each scan was interpreted by two radiologists independently, one having 11 years of experience and the other having 3 years of experience.

### Statistical analysis

Data will be presented as numbers and percentages. Agreement between a radiologist and the computed program will be done in the form of number and site of lesions. Kappa agreement will be calculated with the overall agreement (%). Kappa will be interpreted as a week agreement till 0.3, moderate agreement till 0.6 and strong agreement for more than 0.6. *P* value will be significant at ≤ 0.05.

## Results

This study was conducted to offer a software computer program that will be programmed to detect the radiological CT findings on CT cuts to help radiologists in rapid detection of COVID-19 pneumonic criteria.

The patient’s ages ranged from 12 to 69 years with the mean age among the participants being 39.7 ± 13.5 years. The females represent most of the study population (72.6%), while the male’s percentage was 27.4%.

The studied participants were 73 patients with COVID-19 pneumonia with 154 affected lobes due to the presence of more than one lesion in some patients (as shown in Table 1).

**Table 1:** Characteristics of the lesion among studied participants

Lesion characteristics	Values (no = 154)
*Number of lesions*	
Mean ± SD	2.66 ± 2.50
Range (min–max)	(1.00–12.00)
Median	2.00

Site	Number (%)
Lt lower lobe	44 (28.6)
Lt upper lobe	28 (18.2)
Rt lower lobe	45 (29.2)
Rt middle lobe	15 (9.7)
Rt upper lobe	22 (14.3)

Table 1 shows that the median number of lesions was 2 among the studied participants ranging from 1 to 12 lesions. The most common affected site of the lesions was the lower lobes either in the left side (28.6%) or in the right side (29.2%) followed by the left upper lobe (18.2%), then the right upper lobe (14.3%) and then the right middle lobe (9.7%).

**Fig. 3:**
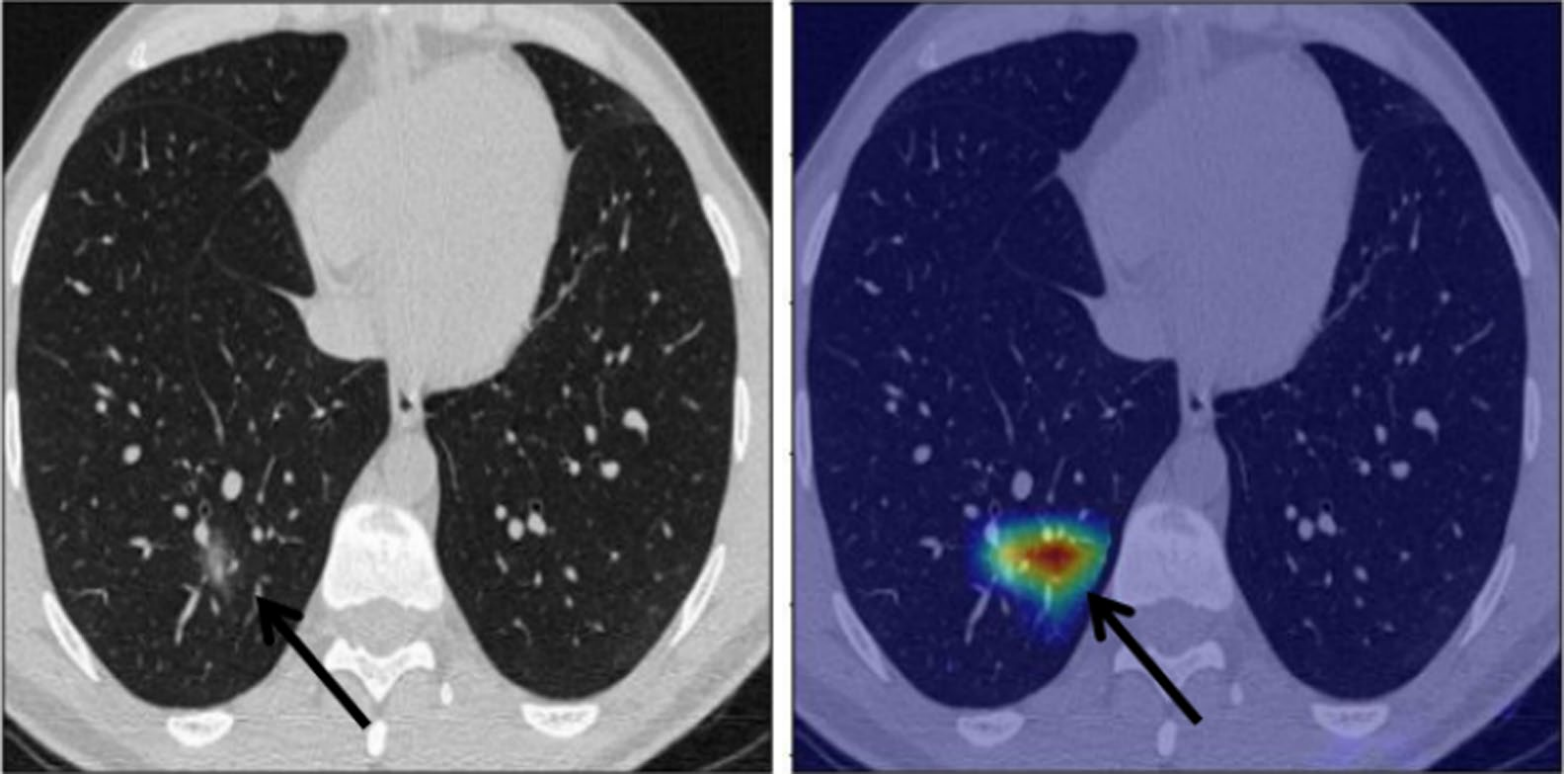
A 34-year-old male patient presented with COVID-19-positive PCR test and suffering from cough and chest pain. CT chest was performed with color-coded images showing left lower patchy of ground glass opacity which was better visualized in color-coded image

There was a significant strong agreement (*P* value < 0.001, kappa = 0.923) between the radiologist and the semiquantitative CT assessment in the detection of GGO among patients with COVID-19 pneumonia (Fig. 3).

**Fig. 4:**
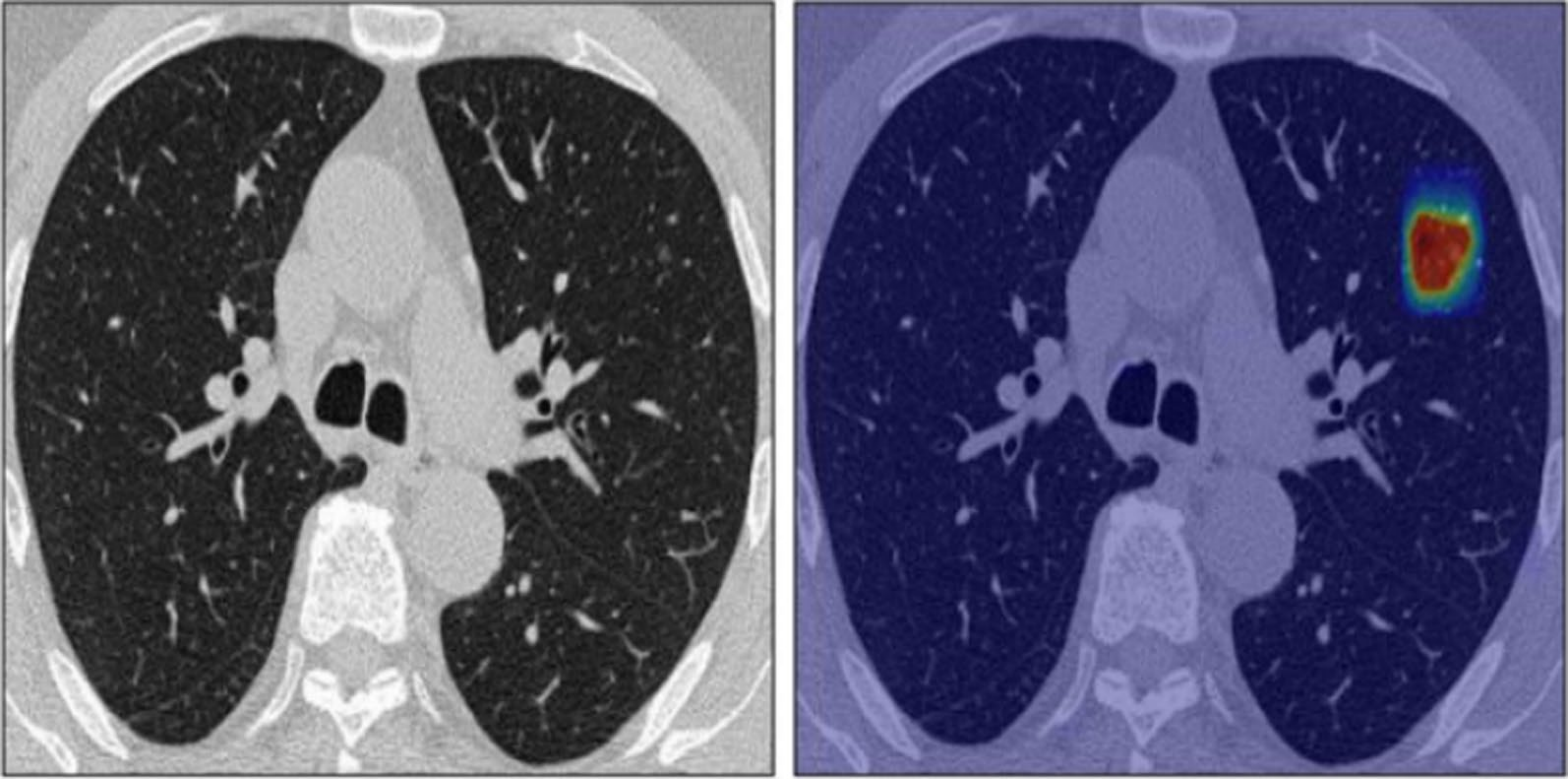
A 67-year-old male patient presented with fever and dry cough. CT chest study was initially unremarkable and after doing color-coded images an ill-defined left upper lobar faint small ground glass nodule became more obvious

**Fig. 5:**
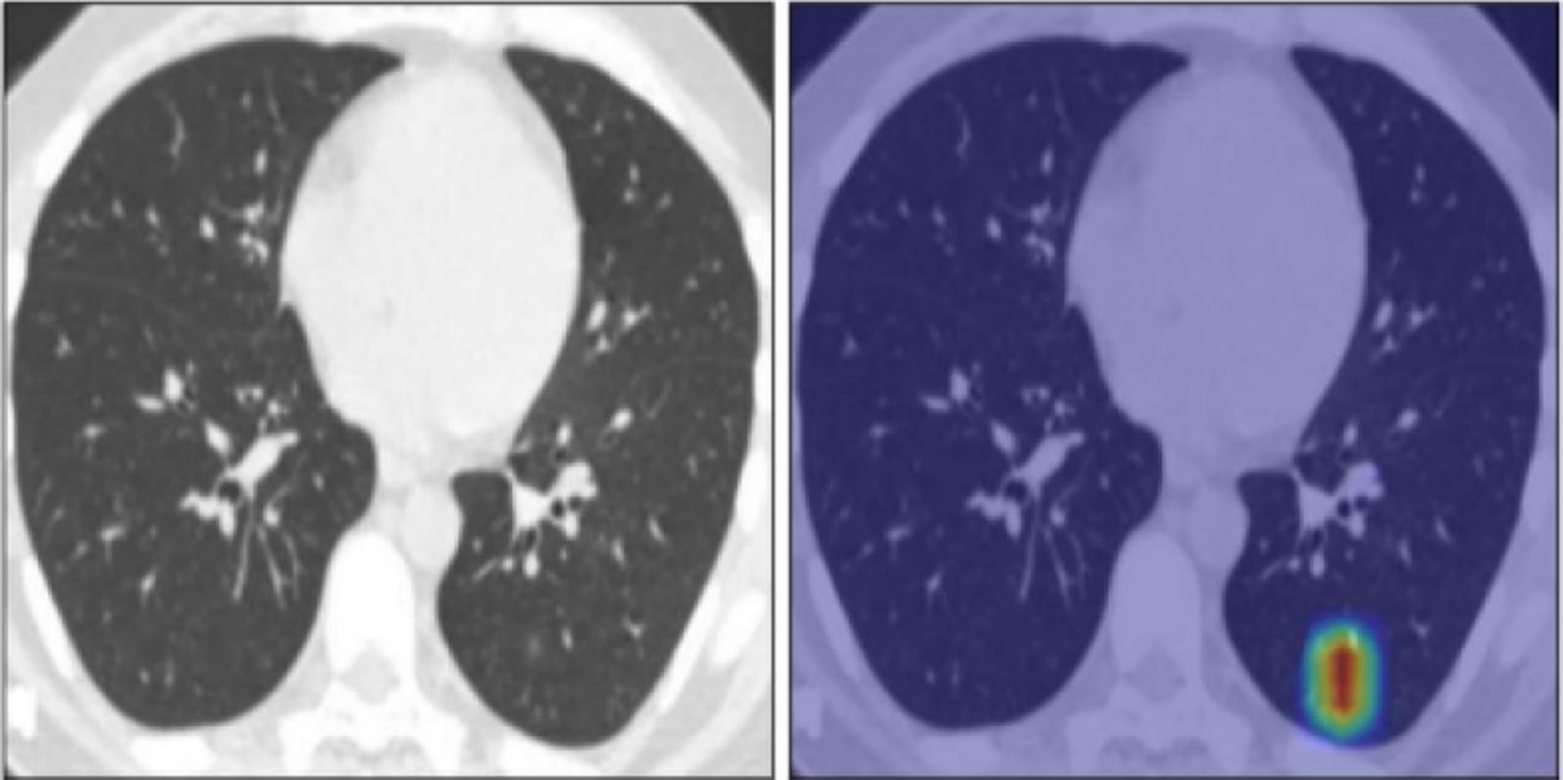
A 36-year-old male patient presented with cough, chest pain and bone ache, yet CT chest was unremarkable and after doing color-coded images an ill-defined left lower lobar faint small ground glass nodule became more evident

The radiologist failed to detect only 9 (5.8%) lesions in 7 patients only. The false negative findings detected by the radiologist were prevalent in the left upper lobe (4 lesions) followed by the left lower lobe (2 lesions) and left lower lobe (2 lesions) (Figs. 4 and 5).

There were 6 patients only who underwent follow-up by semiquantitative CT and radiologist; the 6 patients had 9 affected lobes (Table 2).

**Table 2:** Characteristics of the lesion among studied participants during follow-up (no = 6 patients and 9 lobes)

Lesion characteristics	Values (no = 9)
*Number of lesions*	
Mean ± SD	2.22 ± 2.20
Range (min–max)	(1.00–8.00)
Median	1.00
*Site (no. %)*	
Lt lower lobe	3 (33.3%)
Lt upper lobe	1 (11.1%)
Rt lower lobe	4 (44.4%)
Rt upper lobe	1 (11.1%)

Table 2 shows that the median number of lesions was 1 among the studied participants ranging from 1 to 8 lesions. The most common affected site of the lesions was the lower lobes either in the left side (33.3%) or in the right side (44.4%) followed by the upper lung lobes (11.1% for each upper lobe).

**Fig. 6:**
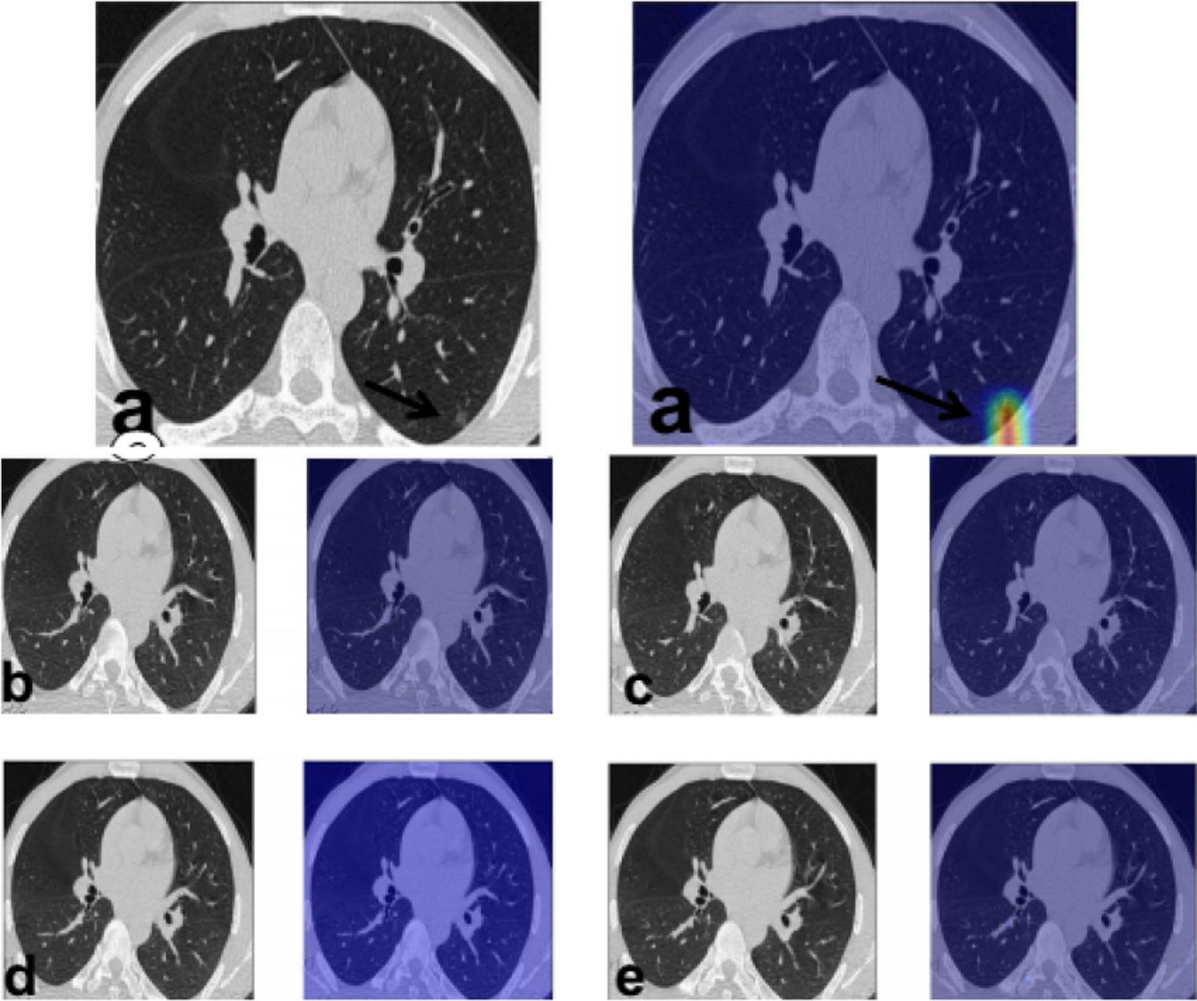
A 34-year-old male patient presented with high grade fever and chest symptoms. **a** MDCT chest was performed revealing a subpleural patchy area of faint ground glass attenuation at the left lower lung lobe which was more prominent at the color-coded images. **b**–**e** Follow-up after 1 month at the same cut level and downward cuts and it appears free at MDCT and better visualized by color-coded images

There was a significant strong agreement (*P* value = 0.001, Kappa = 0.856) between the radiologist and the semiquantitative CT assessment in the detection of GGO during follow-up of the patients with COVID-19 pneumonia (Fig. 6). The radiologist failed to detect only 1 (11.1%) lesion in one patient only.

## Discussion

Chest CT plays an important role in the assessment of patients with positive COVID-19 infection and their follow-up. These examinations are vital in the early detection and assessment of the COVID-19 disease course [8]*.*

CT study of the chest plays a vital role in the evaluation of the severity of COVID-19, monitoring progression of the disease and consequent evaluation of the therapeutic efficiency [9].

At first, confirmed cases of COVID-19 by PCR underwent CT chest study; then, the obtained CT chest cuts were introduced to a software computer program using deep learning and programming to enable the software to make auto-detection of lesions when future CT cuts are processed.

In this study, the median number of lesions detected in the study population was 2 lesions ranged from 1 to 12 lesions. The most common affected site of the lesions was the right lower lung lobe (29.2%), left lower lung lobe (28.6%), followed by the left upper lung lobe (18.2%), the right upper lung lobe (14.3%) and at the last the middle lung lobe (9.7%).

In agreement with the current study results, Bao et al. [10] performed a systematic review and meta-analysis of the results obtained from published studies to provide a summary of the detection of COVID-19 by CT chest study. It was found that the most commonly affected lobe was the right lower lobe (87.21%). Also, Salahshour et al. [11] reported in their study that the most commonly affected lobe is the right lower lobe (43.3%).

According to the current study results, it was found that there was a significant strong agreement (*P* value < 0.001) between the radiologist and the semiquantitative CT assessment in the detection of GGO among patients with COVID-19 pneumonia. The radiologist failed to detect only 9 (5.8%) lesions in 7 patients only. The sites of false negative GGO findings detected by the radiologist in the present study were most prevalent in the left upper lobe (14.3%) followed by the left lower lobe (4.5%) and left lower lobe (4.5%).

In agreement with our results, a study conducted by Pan et al. [12] aimed to explore a novel deep learning-based quantification and compare its efficacy with the conventional semiquantitative CT scoring for the serial chest CT scans of patients with COVID-19. They found that there is a good correlation between conventional CT scoring and novel deep learning-based quantification (*P* < 0.001).

Shan et al. [13] in a study evaluated the overlap ratio (Dice similarity coefficient) between an automatically segmented infection region (*S*) and the corresponding reference region (*R*) provided by radiologist(s). It was found that the average Dice similarity coefficient is 91.6% ± 10.0%.

In a previous study conducted by Wu et al. [14], it was found that the proposed diagnosis model trained on multi-view images of chest CT images, based on the deep learning method, showed great potential to improve the diagnosis and reduce the heavy workload on radiologists in the initial screening of COVID-19 pneumonia.

As regards the patients who were followed up by semiquantitative CT and radiologist in this study population, it was found that the median number of lesions was 1 ranging from 1 to 8 lesions. The most common affected site of the lesions was the lower lobes (right lower lobe 44.4% with 33.3% for the left lower lobe) followed by both upper lobes (11.1% for each lobe).

According to the current study findings, it was reported that there was a significant strong agreement (*P* value = 0.001) between the radiologist and the semiquantitative CT assessment in the detection of GGO during follow-up among patients with COVID-19 pneumonia. The radiologist failed to detect only 1 (11.1%) lesion in one patient only.

In agreement with these results, Gieraerts et al. [15] conducted a study to compare the prognostic value of detection of lung involvement by the visual versus artificial intelligence (AI)–assisted analysis at chest CT in patients with COVID-19 pneumonia and found that there is an average agreement between them.

However, a study conducted by Li et al. [16] reported different results. Their study results showed the superiority of AI-assisted quantification over conventional CT severity scores for the prediction of disease progression. Also, Kimura et al. [17] also concluded that the AI-calculated CT severity score and total opacity percentage showed higher diagnostic accuracy.

Our study had some limitations, first of them is the limited number of patients in the study and moreover the limited number of patients who performed follow-up CT scan. That’s why we recommend to perform future studies on a larger scale of population.

## Conclusions

Lastly, in this study we concluded that the tested computer program can accurately detect COVID-19 pneumonia as it has better visualization in detecting GGO for diagnosing and following up on COVID-19 pneumonia. Semiquantitative technique, based on both visual and color-coded images, helps in improving the early detection of lung affection in COVID-19 patients and consequently helps in the control of infection spread.

## Data Availability

The datasets used and/or analyzed during the study are available upon reasonable request.

## Abbreviations

COVID-19: Coronavirus disease 2019
SARS-CoV-2: Severe acute respiratory syndrome coronavirus 2
GGO: Ground glass opacity
CT: Computed tomography
HU: Hounsfield unit
kV: Kilovoltage
mA: Milliampere
SD: Standard deviation
QIA: Quantitative imaging analysis
NCP: Novel coronavirus pneumonia
AI: Artificial intelligence
